# Improvement in positional accuracy with integrated surface- and X-ray imaging for intracranial stereotactic radiosurgery patients

**DOI:** 10.1016/j.phro.2026.100902

**Published:** 2026-01-09

**Authors:** Caisa Kjellström, Tobias Pommer, Peter Siesjö, Sofie Ceberg, Per Munck af Rosenschöld

**Affiliations:** a Medical Radiation Physics, Lund University, Lund, Sweden; b Radiation Physics, Department of Hematology, Oncology, and Radiation Physics, Skåne University Hospital, Sweden; c Department of Neurosurgery and Section of Neurosurgery, Department of Clinical Sciences, Lund University and Skåne University Hospital, Sweden

**Keywords:** Radiotherapy, Cranial radiosurgery, SGRT, IGRT, SRS, SRT, Motion management, Brain metastasis, Multiple brain metastases, Infra-fractional motion, Intrafraction motion

## Abstract

**Background and purpose:**

Stereotactic radiosurgery (SRS) requires high positional accuracy to safely deliver large doses. This study evaluated an integrated surface- and image-guided radiotherapy (SGRT-IGRT) system by analysing (1) the agreement between thermo-optical and stereoscopic X-ray positioning, and (2) the impact of intra-fractional workflows on treatment accuracy and time.

**Materials and methods:**

Data from 126 SRS patients treated with 30 Gy/3 fractions (n = 116) or 12 Gy/1 fraction (n = 10) on a Varian Truebeam STx were retrospectively analysed. Patients were positioned and monitored with Brainlab ExacTrac Dynamic, with 0.5 mm/0.5° tolerances for IGRT and 1 mm/1° for SGRT. Three workflows were investigated: (A) *SGRT + IntraArc IGRT* (imaging every 90° during treatment and between couch rotations); (B) *SGRT + InterArc IGRT* (imaging between couch rotations only); and (C) *SGRT* (no additional imaging after initial coplanar setup). Workflows (B) and (C) were simulated by omitting applied couch corrections.

**Results:**

Median beam-on times were 5.5 min for workflow A, 5.0 min for workflow B, and 3.2 min for workflow C. The median differences between thermo-optical and stereoscopic X-ray patient positioning were ≤0.1 mm. The 3D positioning uncertainty remained within 0.5 mm (2.5th-97.5th percentile) using SGRT-IGRT. Omitting inter-arc imaging increased positional deviation ranges from 0.1-0.5 mm to 0.1–0.7 mm.

**Conclusion:**

Thermo-optical and stereoscopic X-ray imaging showed good agreement within the set institutional tolerances. Inter-arc imaging increased treatment time by 2 min compared with SGRT alone but improved positioning accuracy. Intra-arc imaging added an additional small accuracy benefit at minor time cost.

## Highlights

• Positioning variation was ≤0.5 mm between surface and X-ray guidance.
• An overall treatment workflow of 10 min was feasible.
• Frequent X-ray imaging reduced large (2–3 mm) positioning variations.
• Image guidance reduced positional variations for non-coplanar fields.

## Graphical abstract

**Figure:**
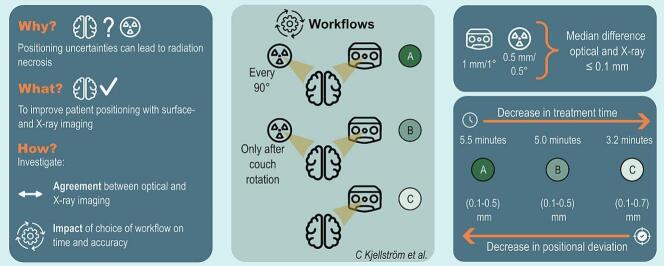

## 1 Introduction

Stereotactic Radiosurgery (SRS) is commonly used to treat intra-cranial metastasis, along with surgery and whole-brain radiotherapy [1]. Delivering a high dose to a small target with steep dose gradients, SRS can achieve good local control [2] and limit side effects through sparing of surrounding normal tissue [3], [4]*.* Despite the high dose conformity of this treatment method, radiation-induced necrosis (RN) with cerebral oedema are common side effects [3]. Pseudoprogression appears early and is related to treatment effect. Signs of RN can occur anywhere from 4-6 months to several years post treatment. If symptomatic, pseudoprogression and RN can manifest as signs of elevated intracranial pressure as headaches, nausea, seizures, but also focal neurologic signs depending on the damaged area of the brain [3]*.*

The risk of RN and oedema increases as the irradiated volume increases, particularly the high-dose volume [3]*.* To enable smaller treatment margins, thus decreasing the total irradiated volume, patient positioning and motion management is crucial. X-ray based image-guided radiotherapy (IGRT) is advantageous for brain SRS due to the skull being a rigid surrogate for target positioning [5], [6]*.* Image guidance can be used in combination with frameless masks, in contrast to the more invasive frame-based systems, to facilitate adequate patient positioning [7], [8], [9]. A more recent patient positioning innovation involves the use of optical light to visualise the patient’s surface [10], [11], [12], [13], so-called surface-guided radiotherapy (SGRT).

Real-time monitoring of the patient using SGRT could potentially reduce the need for continuous X-ray verification and/or fixation devices. In addition, SGRT may reduce or remove the need for fast SRS delivery to maintain good accuracy as was shown by Tarnavski et al. [14]. The feasibility of combined surface and X-ray motion management for SRS was shown previously, indicating that the methodology could be advantageous [15]*.* Arguably, an open question is if radiographic position verification (e.g. using cone-beam computed tomography (CBCT)) with SGRT will provide similar accuracy to workflows including frequent IGRT. Schöpe et al. [16] argues strongly for the need of IGRT during SRS to maintain high accuracy, and the risk of 5–6 mm positional deviations occurring by solely using SGRT for position monitoring [17].

A direct comparison between the workflows of SGRT and IGRT used in a larger patient cohort is currently lacking. Further, uncertainty measures for SRS workflows with a combination of SGRT and IGRT might help clinical teams to be aware of those uncertainties and allow for estimation of appropriate planning target volume (PTV) margins. Therefore, the purpose of this study was to investigate different SGRT and IGRT workflows, ultimately investigating intrafractional motion management on a more granular level in a larger patient cohort.

## 2 Materials and methods

### 2.1 Inclusion

A total of 140 patients consecutively treated with SRS to 30 Gy in three fractions or 12 Gy in one fraction from October 2022 until April 2024 were retrospectively enrolled in this study. Fourteen of the patients were excluded due to data loss, leaving 126 patients (358 fractions, 1186 treatment fields). Of these, 116 patients were treated with 30 Gy in three fractions for primary or secondary brain tumours, and ten patients were treated with 12 Gy in one fraction for benign cranial neoplasms. Clinical variables are presented in Table 1. The median number of targets was 1 (range 1–7). Ethics approval was obtained from the national ethics committee (reference number 2020–04164, Stockholm, Sweden).

**Table 1:** Patient characteristics for the analysed cohort. *N* = 126. For an extended table with listed primary diagnoses, see Table S7 in Supplementary C.

Characteristics	*n*
Sex	
Male	74
Female	52
Age	
<65 years	42
>65 years	78
Histology	
Brain metastasis	114
Benign neoplasms	10
Primary tumour	2
Number of targets	
1	72
2	27
3	11
4	3
5	9
6	2
7	2
ECOG Performance Status scale (WHO)	
Unspecified^a^	35
0	24
1	49
2	12
3	5
4	1

a Some patients did not have performance status noted in the database.

### 2.2 Workflow

A computed tomography (CT) scan with 1.0 mm slice thickness (Siemens SOMATOM Definition AS+, Siemens Healthineers, Erlangen, Germany), and selected T1- and T2-weighted magnetic resonance imaging (MRI) scans, with and without gadolinium contrast, acquired at 1.0 mm slice thickness on a 3 T scanner (GE SIGNA Architect, GE Healthcare, Chicago, IL, USA) were performed on all patients (unless contraindicated) to generate a dose planning basis. Dose planning for cranial SRS were performed in Eclipse (v 15.6, Varian Medical Systems, Inc., Palo Alto, CA, USA). The cohort included both plans optimised with standard volumetric modulated arc therapy (VMAT) (n = 10) and plans optimised with HyperArc (Varian Medical Systems, Inc., Palo Alto, CA, USA) (n = 116). A planning objective restricted the number of monitor units (MU) to 300 MU/Gy for all plans.

Treatments were delivered with 6 MV flattening filter free (FFF) energy (1400 MU/min). The standard beam arrangement consisted of an initial coplanar full arc (358°), or half-arc (180°) combined with 1–3 additional non-coplanar half-arcs delivered at a couch rotation up to ±90° using a single isocentre. All patients were treated on a Varian Truebeam STx linear accelerator (linac) equipped with a multi-leaf collimator with 2.5 mm inner collimator leaves and 5 mm outer collimator leaves, and a couch allowing six degrees of freedom couch corrections.

**Fig. 1:**
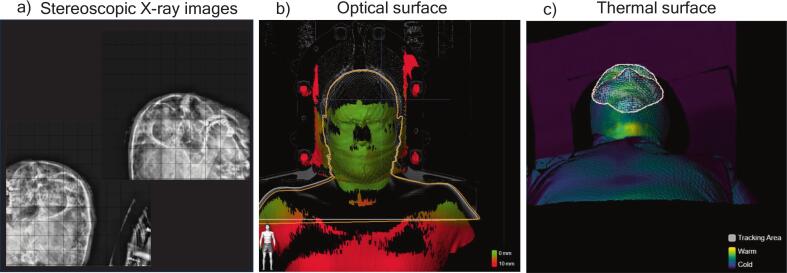
Visualisation of the different system features. A: a pair of stereoscopic X-ray images of a skull. The images are taken from each side of the treatment couch in oblique angles. B: optical surface (green/red). C: thermal information (gradients purple to green) and a marked ROI used for surface scanning (white). (For interpretation of the references to colour in this figure legend, the reader is referred to the web version of this article.)

Patient immobilisation was achieved with either 3-point thermoplastic masks (n = 100, Orfit Industries, Wijnegem, Belgium) or Encompass SRS (n = 26, QFix, Avondale, PA, USA). Patients were set up and monitored with SGRT and IGRT with the Brainlab ExacTrac Dynamic system (v 1.1, Brainlab AG, Munich, Germany). This system integrates a 4D thermal camera (optic surface scanning and thermal surface detection) and ceiling-mounted stereoscopic X-ray imaging system (Fig. 1). An initial surface-based setup was followed by ExacTrac Dynamic stereoscopic X-ray images (hereafter referred to as X-ray images), allowing for corrections of translations (latitudinal, longitudinal, vertical) and rotations (pitch, roll, yaw of ±3°). The region of surface monitoring was manually defined by the radiotherapy technician at the first treatment fraction and includes the majority of the face (Fig. 1C). The defined region was then used for the remainder of the treatment.

After this initial couch correction, an additional pair of X-ray images was acquired, and any residual positioning deviations were corrected. During arc treatment, X-ray images were acquired at gantry angles 0°, 90°, 180° and 270°. However, all non-coplanar arcs were partial, so 1–3 intra-arc images were acquired per treatment field. All intra-arc images were automatically fused to the planning-CT, and any deviations exceeding the tolerances 0.5 mm (3D) or 0.5° (any rotational direction) triggered a beam-hold and subsequent patient repositioning. The live SGRT surface was updated according to recorded patient positional deviations from X-ray images every time new images were acquired. The threshold for surface deviations for repositioning was 1.0 mm (3D) and 1.0° (any rotational direction). Additional X-ray images were acquired after every couch rotation.

### 2.3 Data collection

Position data was collected from the ExacTrac Dynamic software, containing timestamps, MU values, and translational and rotational positional deviations. Additionally, the software recorded whether the datapoint originated from surface tracking (recorded at up to 20 frames per second), X-ray (recorded for every intra-arc image pair) or contained an applied couch shift (recorded in case of beam-hold and patient repositioning). Summary reports containing X-ray deviations between treatment fields from verification imaging were collected and were used in the computations of the simulated workflows.

Overall treatment time (OTT) was collected and was defined as time from first initial setup image to last photon delivery.

### 2.4 Data analysis

The treatment time was extracted from the position data, measured from the first to last photon delivery of the treatment. A beam hold was defined as a timestamp gap in the surface dataset of more than 30 s.

A new reference surface was recorded for every X-ray acquisition, which ensured that the isocentre of both systems were coincident. For each monitoring image acquired during the treatment, the corresponding surface positional deviation was acquired. The 3D motion (*vector_T_*) and total rotation (*vector_R_*) (albeit missing a physical meaning, it was calculated as a metric of a patient’s overall rotation) was computed as the Euclidian norms of translational and rotational vectors, respectively. The difference (*Δp*) was then acquired in all directions and in 3D motion and rotation.

All data analysis was performed in Python version 3.12. The packages Pandas (1.1), SciPy (1.14), and NumPy (2.0) were used.

### 2.5 Modelling imaging frequency

To investigate the effects of varying imaging frequency, three workflows were considered where the latter two were simulated.

A. *SGRT + IntraArc IGRT* – surface scanning and X-ray imaging were used between couch rotations and during treatment delivery. This is the workflow used routinely in our clinic and what the raw data represented.
B. *SGRT + InterArc IGRT* – surface scanning was used throughout delivery, with X-ray imaging only between couch rotations. Simulated by omitting X-ray image triggered couch corrections during beam delivery.
C. *SGRT* – only surface scanning for motion management. Simulated by omitting all X-ray image triggered couch corrections.

**Fig. 2:**
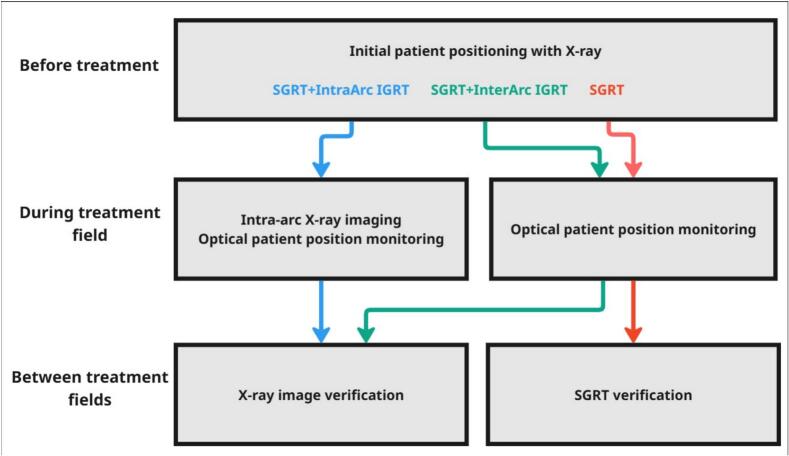
Flowchart overview of the three workflow models simulated in this study: A) *SGRT + IntraArc IGRT*, combining X-ray imaging and thermo-optical motion management between couch rotation and during treatment delivery; B) *SGRT + InterArc IGRT*, uses intrafractional X-ray imaging only between couch rotations, and thermo-optical motion management; C) *SGRT* which only uses thermo-optical motion management.

All workflows started with initial X-ray positioning. An overview of the workflows is presented in Fig. 2, with extended methodology in Supplementary A.

The median positional deviation per treatment field was calculated for each workflow. The proportion of MUs delivered during a set of positional deviation intervals, with increments of 0.1 mm, was calculated for all patients and for the different workflows respectively while separating coplanar and non-coplanar fields. The interval selection was based on the *vector_T_* and *vector_R_* values. In the cases of multiple data points per unique MU, the maximum value of *vector_T_* and *vector_R_* was used.

### 2.6 Statistical analysis

The non-normality of the positional deviation data was tested and confirmed with a Shapiro-Wilk test, excluding parametric tests. The Spearman’s Rank Correlation was used for correlation analysis between image position and surface position datasets, and the Friedman test was used for testing the dataset’s distribution differences. When performing post-hoc tests, pairwise Wilcoxon Signed-Rank test was used. The Z-statistics of the Wilcoxon Signed-Rank test divided by the square root of the sample size was used to calculate effect size. All statistical tests were two-tailed and used an alpha value of 0.05.

In every case where the 95 % Confidence Interval (CI95%) was calculated, it was calculated non-parametrically (as the 2.5 and 97.5 percentile of the dataset) due to non-normality of the data. The outliers in the violin plots represent datapoints outside the interquartile range (IQR). Specifically, these are values outside the range $\left[Q1-1.5*IQR,Q3+1.5*IQR\right]$, where *Q1* (first quartile) and *Q3* (third quartile) define the middle 50 % of the data, and IQR=Q3-Q1. These points were plotted individually to highlight deviations from the main distribution.

Positioning uncertainty, in the form of mean 3D positional deviation for the whole treatment, was investigated in terms of correlation to clinical variables.

For all statistical analysis, Python version 3.12 was used together with the statistics module (1.0.3) and SciPy (1.14) package.

## 3 Results

No significant differences in positional deviation were found between the mask systems (p > 0.1), and all patients were pooled henceforth (Supplementary B.1). The mean OTT for the dataset was 10.5 min (±4.4 min, range 3.8–31.7 min). The mean time between fields with different couch angles was 77.1 s (±59.0 s, range 41–1140 s), which included couch rotation and positional correction with X-ray imaging. The median treatment time was 5.5 min (range: 2.5–13.5, SD: 1.6), measured from the start of the first treatment field to the end of the last treatment field. Most patients were treated using 3 or 4 fields (2: n = 15, 3: n = 53, 4: n = 58). Beam holds (with positional correction) occurred at least once in 56 patients (44 %), and 85 fractions (24 %) had at least one beam hold with positional corrections. A total of 119 beam holds were identified throughout the dataset, averaging 68 s (SD: ± 37 s). Of these, n = 111 (93 %) were caused by the intrafractional X-ray image being out of tolerance, n = 7 (6 %) the surface being out of tolerance (across 4 patients), and n = 1 (1 %) by manual interruption. The number of beam holds decreased in *SGRT + InterArc IGRT* (n = 12) and *SGRT* workflows (n = 50), all caused by the surfaces being out of tolerance. The median treatment time was 5.0 (range: 2.5–13.5, SD: 1.4) minutes and 3.2 (range: 1.8–9.2, SD: 1.0) minutes for the *SGRT + InterArc IGRT* and *SGRT* workflows, respectively.

**Fig. 3:**
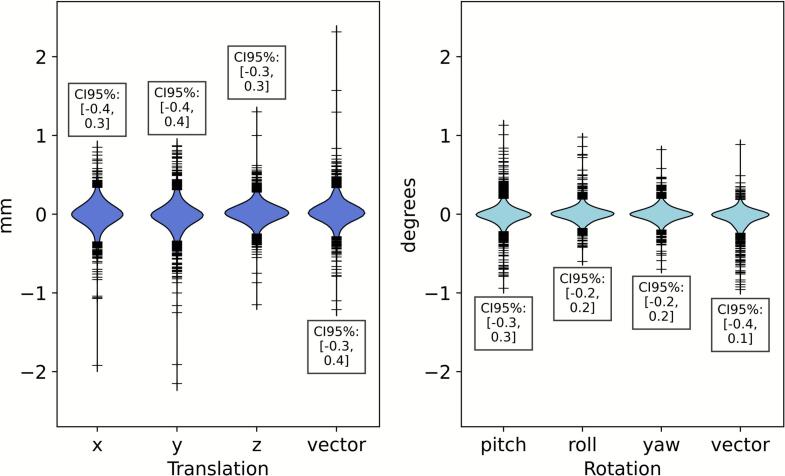
Differences between surface and image patient deviation, shown in violin plots. Directions depicted are *x, y, z, vector (mm) (translational vector), pitch, roll, yaw,* and *vector (deg.) (rotational vector).* Translational and rotational directions are coloured differently for clarity. The median difference for the violins were all ≤0.1 mm and ≤0.1°. Dashes represent outliers.

Among n = 1997 paired surface and image position data points, the median differences were ≤0.1 mm and ≤0.1°, with CI95% limits within ±0.5 mm and ±0.5° (Fig. 3). There were in total two datapoints with >2 mm difference. Significant correlations were observed for all axes (p < 0.001), with weaker correlations in translation (*ρ:* 0.6, 0.5, 0.4, 0.6, *x, y, z, vector_T_* respectively) in contrast to rotation (*ρ:* 0.6, 0.7, 0.5, 0.6 for *pitch, roll, yaw, vector_R_* respectively). Additional results at varying angles of couch rotation can be found in Supplementary B.2.

**Fig. 4:**
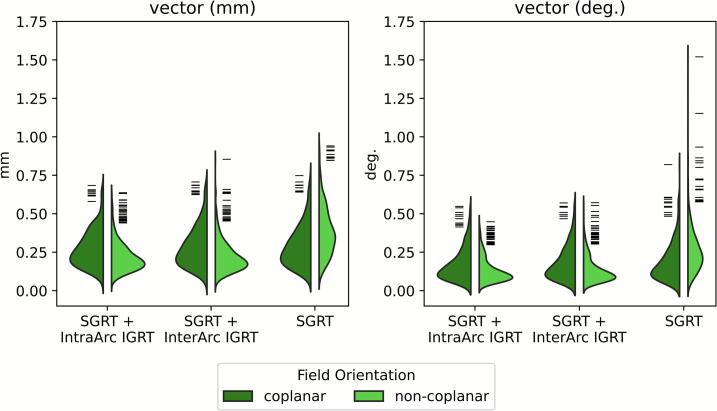
Median patient positional deviation per recorded treatment field across all patients, for the three different workflows *SGRT + IntraArc IGRT*, *SGRT + InterArc IGRT*, and SGRT. Directions depicted are *vector_T_* and *vector_R_;* all other non-vector directions can be found in the supplementary (Fig. S2). Each violin is split into coplanar (left) and non-coplanar (right) fields. Dashes represent outliers.

The median positional deviations per field (Fig. 4) showed smaller CI95% for coplanar fields than for non-coplanar fields across all workflows. For *vector_T_*, CI95% ranged 0.1–0.8 mm, and for *vector_R_*, from ≤0.1° to 0.6°. Workflow comparisons showed significant differences (p < 0.001), with effect sizes ranging from −0.2 to −0.8 depending on field type and axis (detailed statistics in supplement B.3).

A worse ECOG performance score correlated with increased 3D positional deviations across workflows (*ρ:* 0.3, 0.3, 0.3, p < 0.02). See Supplementary B.4 for extended analysis on clinical variables.

**Fig. 5:**
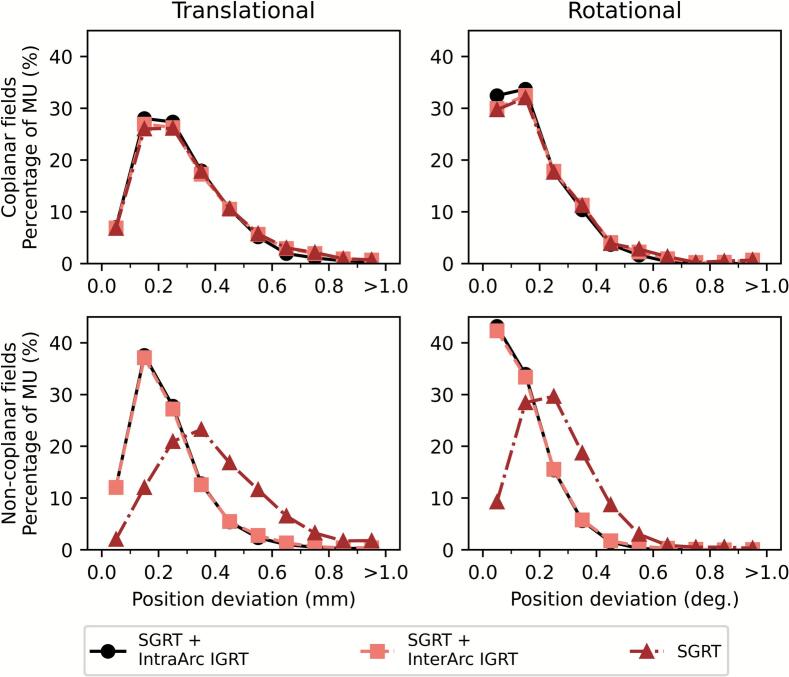
The percentage of total MUs delivered in each defined vector position intervals with 0.1 mm and ° increments. The data is separated into MUs delivered for coplanar and non-coplanar fields and show results from each workflow *SGRT + IntraArc IGRT*, *SGRT + InterArc IGRT*, and *SGRT*. The last interval represents all datapoints above 0.9 mm or 0.9°.

The majority of MUs in coplanar fields were delivered during positional deviations of 0.1–0.2 mm and 0.1–0.2° (Fig. 5), whereas non-coplanar fields in the *SGRT* workflow showed larger deviations (0.3–0.4 mm and 0.2–0.3°) compared to the other workflows.

## 4 Discussion

This study investigated the accuracy and impact of intrafractional stereoscopic X-ray imaging in combination with thermo-optical surface scanning. The two components agreed well, and both intra- and inter-arc X-ray imaging improved patient positioning during treatment delivery for intracranial SRS patients.

The treatment times decreased with reduced X-ray imaging frequency during treatment (p < 0.005), though gains were modest assuming couch correction with X-ray imaging at non-zero angles is possible. Our reported time between treatment fields with different couch angles was shorter than the delays reported by Tarnavski et al. [14], who observed ranges up to almost 30 min with a workflow comparable to the *SGRT + InterArc IGRT* workflow. The reduction in treatment time in our study may also be attributed to workflow improvements in the newer the ExacTrac software, use of VMAT technique and FFF beams. Calmels et al. [18] reported a substantially longer median OTT of 23 min (IQR 20–31 min) with ExacTrac, although using an older version of the system, having comparable delivery techniques to our study. They also reported longer treatment time with ExacTrac than without, consistent with our findings but it is unclear whether the increase occurred at setup or during treatment delivery.

Positional differences between surface and X-ray imaging were in the submillimetre range, but occasional large discrepancies (2–3 mm) justified intra-arc imaging for certain cases. About half of patients had at least one out-of-tolerance X-ray image, lower than Barnes et al. [19] who used 0.7 mm/0.7° tolerances. The difference in reported accuracy across studies [15], [19], [20] may reflect both patient populations and tolerance settings. If intra-arc imaging is to be excluded, the tolerance of the surface imaging system should be reconsidered. Given the high agreement between the two imaging components, and evidence that the thermo-optical camera can detect small positional deviations, the surface tolerance could potentially be reduced to a submillimetre level.

All workflows differed significantly in positioning accuracy (p < 0.001), though the small effect size between intra- and inter-arc imaging suggests limited overall impact in this cohort. However, increased outliers without intra-arc verification indicate some patients may still benefit although the difference was not significant (Kruskal-Wallis test, p > 0.1). Worth mentioning is that the coplanar fields were delivered immediately after initial setup. This allows less time for the patient to shift, in contrast to non-coplanar fields with about a minute for potential patient motion until the next verification image. The *SGRT* workflow showed larger deviations, especially for non-coplanar fields, supporting the role of inter-arc imaging.

A key methodological consideration is the difference in thresholds: 0.5 mm/0.5° for X-ray vs. 1.0 mm/1.0° for SGRT. These thresholds reflect our institutional practise, and this inevitably applies biases to workflows using X-ray toward smaller corrections. While this reflects clinical reality at our institution, it complicates direct workflow comparison and highlights the need for tolerance harmonisation in future studies. Couch rotation and isocentre runout may also contribute to larger discrepancies between IGRT and SGRT in non-coplanar angles, as presented in Supplementary B.2).

A 1 mm/1° tolerance can be used for SGRT in SRS workflows [21], but strategies vary widely: from 0.5 mm/0.5° [18], [22] to 2 mm [23]. Our clinic uses 1 mm/1° in combination with IGRT (0.5 mm/0.5°), but further optimisation of SGRT thresholds alongside IGRT may improve efficiency without compromising precision.

Several studies [16], [20], [22], [23], [24] highlight the value of intra-arc imaging, both due to large position corrections in many studied patients, and possible facial motion-induced uncertainties in optical surface systems. While open masks may risk motion beneath fixation [21], [25], intracranial SRS studies show comparable stability to closed masks [26], [27], with the added benefit of comfort. Despite use of a closed thermoplastic mask, facial structures such as the nose and chin are visible in the thermal view.

There are however limitations with the study. The simulated couch shifts assigned the positional deviation to zero, as did “undoing” the surface update, when in reality noise or imperfect couch positioning is present. The extent of this noise has not been investigated in the current study. These estimations create an optimistic estimate of the accuracy of the surface scanning system, which can effectively underestimate positional deviations when using the SGRT-system. In the future, this underestimation should be investigated further to improve the current model. Isocentric discrepancies should be considered as well, both between the treatment machine and ExacTrac system (up to 0.1 mm), and between the treatment gantry, couch, and collimator (estimated up to 0.5 mm). Whether this may affect the X-ray accuracy across all positions is not explored in this study.

In conclusion, an integrated SGRT and IGRT system improved positional accuracy for intracranial SRS. Thermo-optical and X-ray imaging showed strong agreement within the applied tolerances. Inter-arc imaging was most impactful, particularly in non-coplanar fields. The optimisation of SGRT-IGRT tolerances warrants further study.

## Declaration of Generative AI and AI-assisted technologies in the writing process

During the preparation of this work the author(s) used ChatGPT v4o in order to improve language and readability in selected sentences. After using this tool/service, the author(s) reviewed and edited the content as needed and take(s) full responsibility for the content of the publication.

## CRediT authorship contribution statement

**Caisa Kjellström:** Writing – original draft, Data curation, Formal analysis, Investigation, Methodology, Visualization. **Tobias Pommer:** Writing – review & editing, Supervision, Conceptualization, Methodology, Validation. **Peter Siesjö:** Writing – review & editing, Supervision, Conceptualization. **Sofie Ceberg:** Writing – review & editing, Supervision, Conceptualization. **Per Munck af Rosenschöld:** Writing – review & editing, Funding acquisition, Supervision, Conceptualization, Resources, Project administration.

## Declaration of competing interest

The authors declare the following financial interests/personal relationships which may be considered as potential competing interests: P. Munck af Rosenschöld, together with the Radiotherapy Clinic of Skånes University Hospital, has a research agreement with Brainlab AG, Munich, Germany, and Accuray Inc., Madison, WI, USA. P. Siesjö is an external consultant for Lantmännen Medical AB, Stockholm, Sweden, and for Clinical Laster Systems AB, Lund, Sweden. Remaining authors have nothing to declare.
