# Supplementary material for: Improvement in positional accuracy with integrated surface- and X-ray imaging for intracranial stereotactic radiosurgery patients
Source: Phys Imaging Radiat Oncol. 2026 Jan 9;37:100902. doi: 10.1016/j.phro.2026.100902 (PMC12828801; doi:10.1016/j.phro.2026.100902)
Supplement: Supplementary Data 1 [file mmc1.pdf]

Supplementary

A. Expanding the method of workflow model simulations

To model workflow 2 and 3, the applied couch shift corrections had to be removed. Simulating this required the following assumptions:

- The X-ray images acted as the reference in this study and patient positional deviations calculated from them were assumed to be the most correct positional deviation.

- The patient was assumed to keep the same motion fluctuation in the simulated workflows as they had during the recorded treatment session.

- Uncertainties concerning noise are omitted in this model, as it was aimed to represent the patient position and not the performance of the SGRT system.

With these assumptions, the following computations were made:

- When an image was taken and a couch shift was applied, the surface was updated, and the patient and couch was shifted to accommodate the calculated deviation. To remove this event, the patient position without the applied couch shift $\hat{p}$ was instead taken to be the calculated positional deviation acquired from the image p_{acs}. It was then assumed to vary with the same motion pattern *m* until next event of interest (image or surface out-of-tolerance deviations), resulting in the following computation:

  $$\hat{p} = p_{ACS} + m \quad (S1)$$

  $$m = p_0 - p_n \quad (S2)$$

  where *p* is the array of surface datapoints that follow the imaging, p_0 is the first element in *p*, and p_n is the n:th element in *p*. This results in *var* being an array as well. This was applied on images during treatment for *SGRT+InterArc IGRT* and *SGRT* workflows.

- Image patient positional deviations were originally acquired in the treatment workflow where couch shifts were applied during and between the treatment fields (raw data). Because there is no X-ray image correction between the fields in the *SGRT* workflow (i.e. the workflow only relies on the thermo-optical imaging, which can still trigger a positional correction if the deviation exceeds the tolerance of 1 mm (3D) or 1° (any rotational direction)), there is an additional deviation that needs to be accounted for in the simulation. This additional deviation, denoted as δ, originates from the simulated patient position at the end of the previous treatment field. In the absence of positional correction in previous treatment fields, the resulting simulated verification image takes this positional shift into account. The δ thus represents a continuation of the patient's relative motion pattern, based on the assumption that intra-fractional motion fluctuations remain consistent between the simulated workflows.

- The δ was calculated as the difference between the last surface positional deviation in the previous field of the raw dataset and the verification image deviation. The δ is added to the starting point of the subsequent field, effectively representing the image positional deviation that would have been observed between couch rotations.

  $$\delta = p'_{last,surface} - p_{verification,image} \quad (S3)$$

- If any simulated surface point was out of tolerance for at least the reaction time of one second, a simulated beam hold will occur. Because the timestamps did not have the precision needed, number of data points per second was used instead. A mean reaction time across all recorded treatment beams of fifteen points per seconds was acquired. After the simulated beam hold, equation S1 was used with $p_{ACS} = 0$ assuming the couch was moved according to the deviations from the origin.

- Each time a beam hold caused by an image occurred, the time stamps were collected. The beam hold time was subtracted from the total treatment time. If there were simulated beam holds in the *SGRT+InterArc IGRT* and *SGRT* workflows, time equal to number of simulated beam holds in the treatment fraction multiplied with mean beam hold time was added to the treatment time.

- Additionally, for the SGRT workflow the extra time caused by X-ray imaging had to be removed. Timestamps of these images were not present in the dataset, so it could not be subtracted from the recorded time. Instead, couch rotation times were measured physically at the linear accelerator. Data points included rotations starting at 0° rotating to the angles present in the dataset (270°, 315°, 310°, 20°, 45°, 50° and 90°) and rotations in between some of these. From this data, a linear function was fitted to calculate time per rotated angle. This was then applied between the fields of the SGRT workflow model. An uncertainty of about a second needs to be considered in this case.

B. Additional results

B.1 Statistical tests between mask types

There existed two types of immobilisation masks in the dataset – 3-point thermoplastic masks (Orfit Industries, Wijnegem, Belgium) and Encompass SRS (QFix, Avondale, Pa, USA). Mann-Whitney U tests were performed on arrays of mean positional shifts per 1) treatment field, 2) treatment fraction, and 3) total treatment between the two mask groups. No significant differences were found (p > 0.1) (Table S1).

Table S1: Table of Mann Whitney U p-values resulting from test between Orfit and Encompass group patient positional shift in all directions. There were three aggregations; mean per treatment field, mean per treatment fraction, and mean per treatment. Number of instances per group is presented at the bottom of the table.

	Mann-Whitney U p-value		
Aggregation	Mean per field	Mean per fraction	Mean per treatment
x	0.96	0.85	0.72
y	0.83	0.91	0.90
z	0.86	0.87	0.92
pitch	0.39	0.46	0.96
roll	0.54	0.90	0.85
yaw	0.75	0.61	0.57
N Encompass	264	78	26
N Orfit	992	280	100

B.2 Extension on IGRT-SGRT difference

The difference in recorded patient position between the IGRT and SGRT system was significantly altered in only two directions (y, z) and total rotation (figure S2). All mean values were <0.1 mm/0.1° and standard deviations were <0.25 mm/0.25° (Table S1). The ρ of the medium group (20°, 45°, 50°, 315°, 310°, 340°) was generally lower than that of the other angular groups (Table S2).

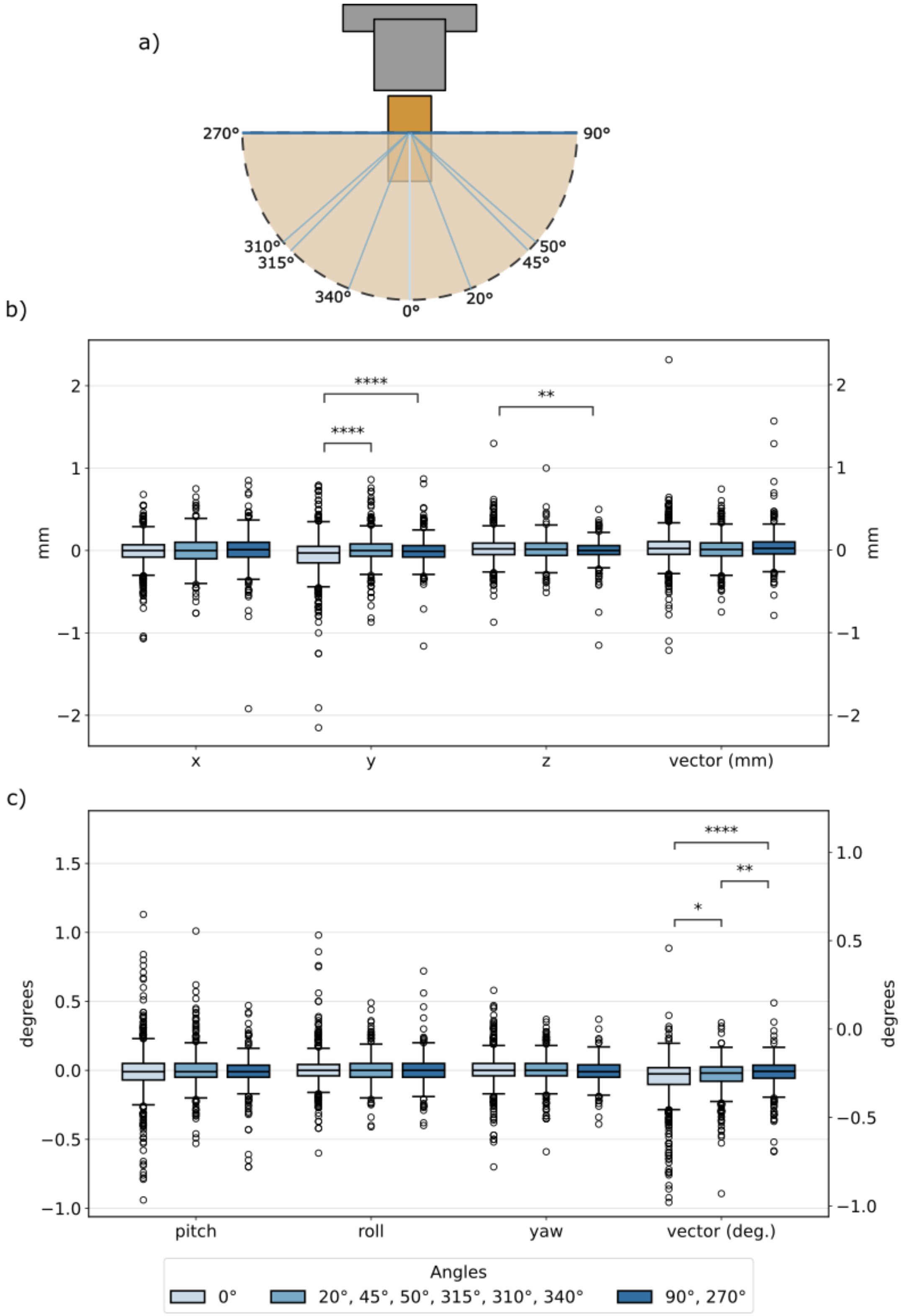

Figure S1: a) Figure visualising the angles that were present in the dataset. The coordinate system used was IEC61217 CCW359.
The boxplots show, similar to Figure 3 in the manuscript, the difference in recorded patient position between the IGRT and SGRT system components in b) translational and c) rotational directions in varying angular groups. Brackets above selected boxes represent significance (* = <0.05, ** = <0.01, *** = <0.001, **** = <0.0001).

Table S2: Statistics of Figure S2. The angle groups consist of 0°, medium (20°, 45°, 50°, 315°, 310° and 340°), and high (90°, 270°) rotation.

Parameter	*Angle group*	*N*	*Mean*	*STD*	*Min*	*25%*	*50%*	*75%*	*Max*
x	0°	820	-0.01	0.17	-1.07	-0.08	0.00	0.07	0.68
	Medium	691	0.01	0.21	-1.92	-0.08	0.01	0.10	0.85
	High	466	0.00	0.17	-0.76	-0.10	0.00	0.10	0.75
y	0°		-0.06	0.24	-2.15	-0.15	-0.03	0.05	0.79
	Medium		-0.01	0.16	-1.16	-0.08	-0.01	0.06	0.87
	High		0.01	0.17	-0.87	-0.07	0.00	0.08	0.86
z	0°		0.02	0.15	-0.87	-0.05	0.02	0.09	1.30
	Medium		0.00	0.13	-1.15	-0.05	0.00	0.06	0.50
	High		0.02	0.13	-0.51	-0.06	0.02	0.09	1.00
$vector_T$	0°		0.03	0.19	-1.21	-0.05	0.02	0.11	2.32
	Medium		0.04	0.18	-0.79	-0.04	0.03	0.10	1.57
	High		0.02	0.15	-0.75	-0.07	0.01	0.09	0.75
pitch	0°		-0.01	0.17	-0.94	-0.07	-0.01	0.05	1.13
	Medium		-0.01	0.11	-0.70	-0.05	-0.01	0.04	0.47
	High		0.00	0.12	-0.53	-0.05	-0.01	0.05	1.01
roll	0°		0.01	0.12	-0.60	-0.04	0.00	0.04	0.98
	Medium		0.00	0.10	-0.40	-0.05	0.00	0.05	0.72
	High		0.00	0.09	-0.41	-0.05	0.00	0.05	0.49
yaw	0°		0.00	0.11	-0.70	-0.04	0.00	0.05	0.58
	Medium		-0.01	0.08	-0.39	-0.05	-0.01	0.04	0.37
	High		0.00	0.10	-0.59	-0.04	0.00	0.05	0.37
$vector_R$	0°		-0.06	0.15	-0.96	-0.10	-0.03	0.02	0.89
	Medium		-0.02	0.10	-0.59	-0.06	-0.01	0.04	0.49
	High		-0.04	0.11	-0.89	-0.08	-0.02	0.02	0.35

Table S3: Spearman correlation in recorded patient position between IGRT and SGRT system. The angle groups consist of 0°, medium (20°, 45°, 50°, 315°, 310° and 340°), and high (90°, 270°) rotation.

Parameter	*Angle Group*	*ρ*	*P*
x	0°	0.64	<0.001
	Medium	0.47	<0.001
	High	0.50	<0.001
y	0°	0.52	<0.001
	Medium	0.50	<0.001
	High	0.56	<0.001
z	0°	0.48	<0.001
	Medium	0.38	<0.001
	High	0.43	<0.001
$vector_T$	0°	0.52	<0.001
	Medium	0.32	<0.001
	High	0.40	<0.001
pitch	0°	0.60	<0.001
	Medium	0.43	<0.001
	High	0.58	<0.001
roll	0°	0.64	<0.001
	Medium	0.53	<0.001
	High	0.54	<0.001
yaw	0°	0.73	<0.001
	Medium	0.54	<0.001
	High	0.68	<0.001
$vector_R$	0°	0.61	<0.001
	Medium	0.49	<0.001
	High	0.56	<0.001

B.3 Extension of figure 4

The remaining results from figure 4 can be found in figure S2. The trends are similar between the two figures, with increasing (albeit non-significant, $p>0.05$) number of outliers between the *SGRT+IntraArc IGRT* and *SGRT+InterArc IGRT* workflows, and larger positional deviations in coplanar fields in the *SGRT* workflow. The CI95% belonging to each violin in figure S2 is presented in table S3. Because the effect sizes were discussed briefly in the discussion, the effect sizes of the remaining 2D directions as well as 3D directions are listed in table S4. Where no significant difference was found, the table cells were left blank.

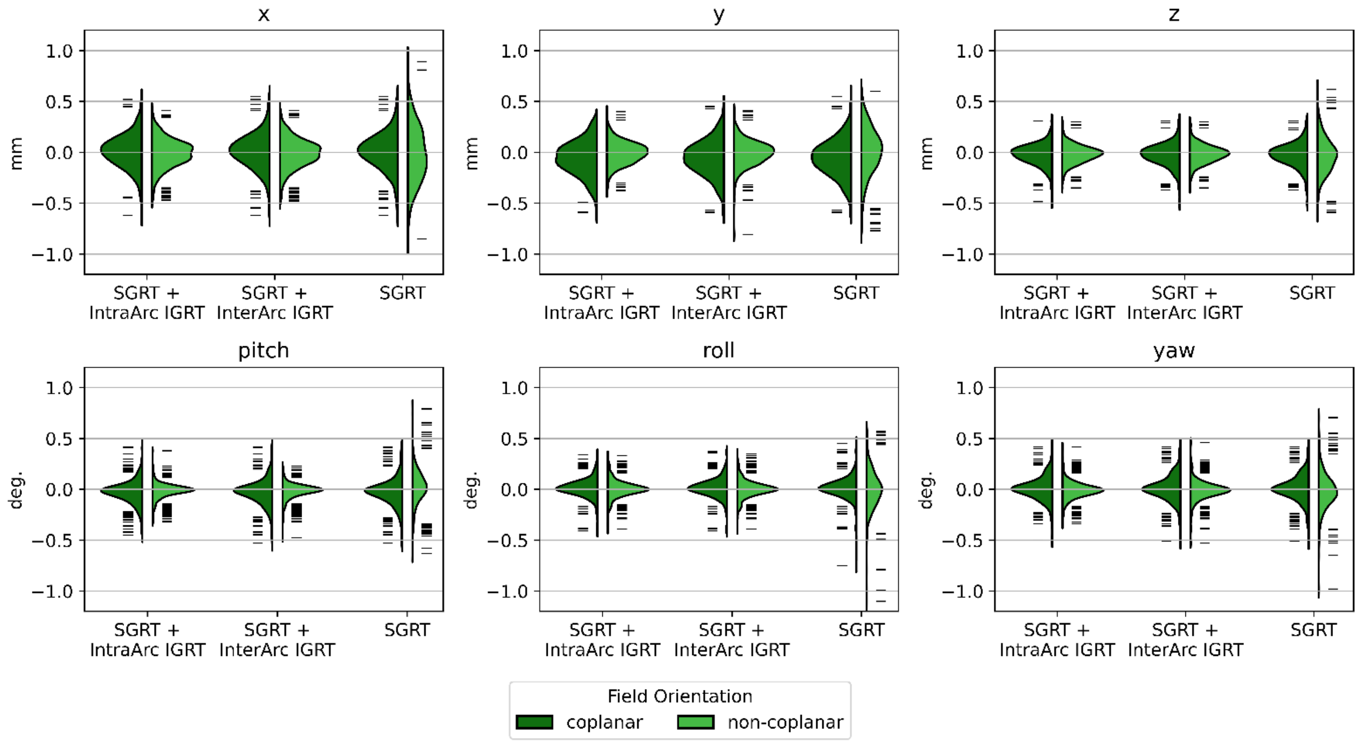

Figure S2: Distributions of median patient positional deviation per recorded treatment field across all patients, for the three different workflows *SGRT+IntraArc IGRT*, *SGRT+InterArc IGRT*, and SGRT. Directions depicted are all other non-vector directions. Each violin is split into coplanar (left) and non-coplanar (right) fields. Dashes represent fliers.

Table S4: Table of CI95% for the median patient positional deviation in translational and rotational directions, related to the violin plots in figure S1.

	SGRT+IntraArc IGRT		*SGRT+InterArc IGRT*		*SGRT*	
Direction	Coplanar	Non-coplanar	Coplanar	Non-coplanar	Coplanar	Non-coplanar
X (mm)	-0.33—0.33	-0.30—0.26	-0.35—0.35	-0.31—0.26	-0.37—0.35	-0.54—0.51
Y (mm)	-0.40—0.26	-0.22—0.22	-0.41—0.29	-0.23—0.22	-0.41—0.29	-0.48—0.44
Z (mm)	-0.23—0.17	-0.20—0.14	-0.24—0.19	-0.20—0.15	-0.25—0.19	-0.35—0.33
Pitch (°)	-0.27—0.20	-0.19—0.11	-0.28—0.20	-0.19—0.11	-0.30—0.24	-0.34—0.34
Roll (°)	-0.17—0.20	-0.18—0.21	-0.17—0.20	-0.18—0.21	-0.17—0.20	-0.30—0.35
Yaw (°)	-0.19—0.24	-0.18—0.16	-0.22—0.25	-0.18—0.18	-0.28—0.25	-0.33—0.31

Table S5: Table of effect sizes of the Wilcoxon Signed-Rank test statistics between the workflows. Empty cells depicts that no significant difference was found (p>0.05) between the two compared workflows in the field orientation. Here, some of the workflow names are abbreviated for readability; *SGRT+IntraArc IGRT = Intra, SGRT+InterArc IGRT = Inter*.

	Comparison	*x*	*y*	*z*	*vector$_T$*	*Pitch*	*Roll*	*Yaw*	*vector$_R$*
All field orientations	Intra vs Inter	-0.01	-0.03	-0.02	-0.20	-0.05	-	-0.04	-0.23
	Intra vs SGRT	-0.05	-0.09	-0.08	-0.67	-0.23	-	-0.07	-0.69
	Inter vs SGRT	-0.45	-0.10	-0.08	-0.64	-0.25	-	-0.07	-0.66
Coplanar fields	Intra vs Inter	-	-	-0.09	-0.27	-	-	-	-0.33
	Intra vs SGRT	-	-	-0.08	-0.32	-	-	-	-0.37
	Inter vs SGRT	-	-	-0.04	-0.16	-	-	-	-0.15
Non-coplanar fields	Intra vs Inter	-0.01	-0.01	-0.03	-0.15	-0.04	-	0	-0.17
	Intra vs SGRT	-0.05	-0.13	-0.08	-0.78	-0.29	-	-0.08	-0.79
	Inter vs SGRT	-0.05	-0.12	-0.09	-0.77	-0.30	-	-0.08	-0.78

The results from comparing the 3D positional deviation between the workflows can be presented as root mean square (RMS) values. In this case, all patients and fractions were pooled, and the RMS was performed in each separate direction. Results are presented in table S4.

Table S6: Table of root mean squared positional deviation for the whole dataset, in each 2D and 3D directions, and workflows.

	x (mm)	*y (mm)*	*z (mm)*	*vector$_T$ (mm)*	*pitch (°)*	*roll (°)*	*yaw (°)*	*vector$_R$ (°)*
SGRT+IntraArc IGRT	0.19	0.17	0.13	0.29	0.11	0.10	0.11	0.19
SGRT+InterArc IGRT	0.20	0.19	0.14	0.30	0.13	0.11	0.12	0.21
SGRT	0.27	0.24	0.18	0.41	0.17	0.16	0.16	0.28

B.4 3D positioning with clinical variables

On the association of 3D positioning with clinical variables, there was also an association, independent of workflow, between 3D positioning uncertainty and gender (ρ: 0.25, 0.24, 0.25, p=0.017, 0.020, 0.017) with men having larger positional deviation than women. Age and number of targets were non-significant.

C. *Extended patient characteristics table*

Table S7: Detailed overview of diagnoses, as ICD diagnosis codes, within the analysed dataset. * C4A.9 = Merkel cell carcinoma, C76.2 = Abdominal cancer, C23 = Gallbladder cancer, C53.9 = Cervix cancer, D32.0 = Meningioma, C65 = Renal pelvis cancer, C34.1 = Cancer in upper lobe, bronchus or lung, C19 = rectosigmoid cancer

Primary diagnoses (ICD codes)	*n*
C34.9 (Lung cancer)	56
C43.9 (Skin cancer)	18
C64 (Kidney cancer)	11
C50.9 (Breast cancer)	10
D33.3 (Cranial nerve schwannoma)	9
C18.9 (Colon cancer)	7
C25.9 (Pancreas cancer)	3
C20 (Rectal cancer)	2
C71.9 (Brain cancer)	2
C4A.9, C76.2, C23, C53.9, D32.0, C65, C34.1, C19 *	One each
